# The impact of bedtime technology use on sleep quality and excessive daytime sleepiness in adults

**DOI:** 10.5935/1984-0063.20200128

**Published:** 2022

**Authors:** Saad Mohammed AlShareef

**Affiliations:** Imam Mohammad Ibn Saud Islamic University (IMSIU), Department of Medicine, College of Medicine, Riyadh 13317-4233, Saudi Arabia.

**Keywords:** Epworth Sleepiness Scale, Electronic Device, Excessive Daytime Sleepiness, Sleep Hygiene, Smartphone

## Abstract

**Objectives:**

There have only been a few studies on electronic device use and sleep in adult populations, so we sought to investigate the impact of bedtime technology use on sleep quality and excessive daytime sleepiness (EDS) through a population-wide survey of Saudi Arabian adults.

**Material and Methods:**

This cross-sectional survey of 10,106 Saudi Arabian adults gathered data on the number and frequency of electronic device use (smartphones, tablets, computers, televisions, radios, and music players) at bedtime, sleep quality, and EDS as measured by the Epworth sleepiness scale. Associations between electronic device number and frequency of use and sleep-related outcomes were evaluated using binary logistic regression.

**Results:**

Twenty-eight percent and 9.7% of respondents reported “fairly” or “very bad” sleep quality in the preceding month, respectively. 95.1% of respondents had smartphones in their bedrooms, which were used regularly (a few nights a week, every or almost every night) by 80.7% of respondents. The number of devices in the bedroom had little effect on sleep quality parameters and EDS, but regular use of almost all devices was associated with “bad” or “very bad” sleep quality (odds ratios (ORs) 1.32-2.12); smartphone or tablet use was associated with sleep latency >30 minutes (smartphones OR 1.98, 95% CI: 1.51-2.60; p<0.0001; tablets OR 1.44, 95% CI: 1.05-1.99; p<0.05). Electronic device use was associated with a 1.3-1.9-fold risk of moderate to severe EDS.

**Discussion:**

This large study strengthens the limited evidence in adults that electronic device use during bedtime usually reserved for sleep impacts sleep quality. Sleep hygiene advice must be updated to include limiting electronic device use in the bedroom.

## INTRODUCTION

Sleep is essential to human health, wellbeing, and daily functioning, impacting not only the individual’s mental and physical health^1^ but also society. For instance, excessive daytime sleepiness (EDS) is associated with high body mass index (BMI), diabetes mellitus, depression, and reduced quality of life^2,3^, up to a third of fatal motor vehicle accidents are thought to involve sleepy drivers^4^, and sleepiness at work is known to represent a significant economic burden to the individual, healthcare systems, and employers^5^. Sleep and sleepiness therefore have widespread impacts on all aspects of public health and the economy, mandating measures to mitigate the consequences of sleep-related dysfunction. To achieve this, understanding the factors impacting sleep quality at the population level is essential.

Technological advances, increased standards of living, demand for 24/7 professional and personal availability and most recently changing social interactions to web-based communication due to the COVID-19 pandemic have transformed the home environment. This is particularly true in the bedroom, which has in many homes become media-rich, containing multiple electronic devices including smartphones, televisions, tablet devices, and computers. In particular, the advent of the smartphone - through its ubiquity, portability, and connectivity - has made it convenient to use at least one form of electronic device in bed. The constantly evolving and changing nature of technology means that there is an ongoing need to study the impact of electronic devices on sleep behavior to inform policy on sleep hygiene fit for the technological era.

There is now abundant evidence that the use of electronic devices at night can adversely impact sleep behavior, resulting in sleep loss, irregular sleep-wake patterns, poorer sleep quality, and EDS, particularly in children and adolescents^6-12^. Several mechanisms have been proposed as to how electronic devices affect sleep quality: (i) exposure to the bright light emitted by electronic devices, particularly short wavelength (blue) light, can suppress melatonin secretion to delay sleep onset and disrupt sleep^13^; (ii) indirectly, by displacing sleep (i.e., taking up time that would otherwise be spent sleeping)^14^; and (iii) increased arousal (mental, physical, and/or physiological) through the nature of the content, which can often be graphic, violent, emotional, or sexual^15^. However, the majority of current studies on technology use and its impact on sleep have been conducted in children and adolescents, and it is unclear whether the impact of technology use on sleep is the same between this population and the adult population. Indeed, rather than being a predictor or sleep disturbance, technology use might be a consequence of poor sleep in adults^16^. Very few studies on technology use and sleep disturbance have been conducted at the population level in adults, with those that have being of limited sample size. Similarly, most studies on this topic have examined individual devices such as smartphones but not the full range of electronic devices that might be found in bedrooms such as televisions, computers, and tablet computers, while others have only examined specific technology-related behaviors such as social media use^17^.

We therefore sought to investigate the impact of bedtime technology use on sleep quality and EDS by conducting a population-wide survey of adults in Saudi Arabia. Specifically, we investigated: (i) the number and frequency of use of electronic devices (smartphones, tablets, computers, televisions, radios, and music players) in the population; (ii) the prevalence of sleep quality and EDS disturbances; and (iii) the relationships between electronic device use and sleep quality and EDS disturbances.

## MATERIAL AND METHODS

### Participants and methods

#### Population and study survey

This cross-sectional study was conducted online between November 6, 2019 and December 6, 2019, as previously reported^18,19^. Briefly, participants aged 18 years and older were randomly selected from the Saudi Telecom Company (STC) database, which covers all 13 Saudi provinces, and invited to participate by e-mail and telephone. Participants were informed of the research purpose and the investigator details. Each participant provided electronic consent. The internal review board (IRB) of Imam Mohammad Ibn Saud Islamic University (IMSIU) approved the study protocol.

### Study questionnaire

The study survey was a wide-ranging questionnaire designed to establish how sleep quality and EDS impact social functioning and a range of outcomes not limited to the impact of technology use but also other outcomes such as occupational outcomes and motor vehicle accidents, as described elsewhere^18,19^. In addition to including questions on specific social outcomes devised according to literature review, the questionnaire assessed sleep parameters using validated instruments such as the Epworth sleepiness scale. Briefly, the questionnaire (see Supplementary Information for those parts of the questionnaire relevant to the current study) was administered in Arabic and assessed: (i) demographics (gender, age, height, weight, and marital status); (ii) sleep quality (subjective assessment of sleep quality measured as very good, fairly good, fairly bad, very bad; sleep latency measured as 0-5min, 5-15min, 15-30min, or >30min; sleep duration (in hours), and sleep efficiency (proportion of time spent asleep whilst in bed, expressed as a percentage); (iii) the Epworth sleepiness scale (ESS; validated Arabic version)^20^, subcategorized as per Johns (1991)^21^ into 0-10 normal daytime sleepiness, 11-12 mild excessive daytime sleepiness, 13-15 moderate excessive daytime sleepiness, 16-24 severe excessive daytime sleepiness; (iv) the presence or absence of electronic devices (smartphones, tablet computers, music players, computers/laptops, televisions, and radios) in the bedroom; and (v) the frequency of use of these devices (never, rarely, a few nights a month, a few nights a week, every or almost every night) when they should have otherwise been sleeping.

### Outcome measures

Five outcomes were investigated and assessed: (i) hours of sleep; (ii) sleep efficiency (sleep efficiency=total sleep time/time in bed, expressed as a %); (iii) sleep quality (very good, fairly good, fairly bad, or very bad); (iv) sleep latency (0-5min, 5-15min, 15-30min, or >30min); and (v) EDS (normal, mild, moderate, and severe).

### Statistical analysis

Participant demographics were analyzed using descriptive statistics with means (±standard deviation (SD)) for continuous variables and counts (with percentages) for categorical variables. For logistic regression, the number of devices in the bedroom was binarized into ≤1 or ≥2, and frequency of electronic device use was binarized into “infrequent” (never, rarely, or a few nights a month) and “frequent” (a few nights a week, every or almost every night). Binary logistic regression models were built for each outcome variable controlling for age, gender, BMI, marital status, and sleeping medication use. There were no strong intercorrelations between variables, as assessed by pairwise correlations all being ≤0.7^22^. Odds ratios were calculated with 95% confidence intervals (CIs). A *p*-value of <0.05 was considered statistically significant. All analyses were performed using IMS SPSS Statistics v. 24 (IBM Statistics, Chicago, IL, U.S.).

## RESULTS

### Overall sample

A total of 10,106 individuals completed all or part of the survey. The demographics of the survey respondents are presented in Table 1. The average age of respondents was 30.7 (SD±11.3) years, with an average BMI of 26.7 (SD±7.7) kg/m^2^. Most respondents were single (53.6%) or married (42.9%). Thirteen percent of respondents had taken some form of medication to aid sleep in the preceding month, the majority of whom (9.3%) had taken medication only infrequently.

**Table 1:** Demographics of the survey respondents (total n=10,106).

Variable		Number	Mean (SD)		Proportion, %
**Age (years)**		8,617	30.7 (11.3)		
**Gender**	Male	4,089		47.3	
	Female	4,560		52.7	
**BMI (kg/m^2^)**		8,602	26.7 (7.7)		
**Marital status**	Married	3,699		42.9	
	Divorced	254		2.9	
	Single	4,616		53.6	
	Widowed	48		0.6	
**Sleeping medications**	Never	7,550		74.7	
	Several days	941		9.3	
	More than half of days	208		2.1	
	Nearly every day	160		1.6	

### Prevalence of sleep quality parameters and daytime sleepiness

Overall, the prevalence of poor sleep quality was high in the study population, with 38% of respondents reporting “fairly bad” or “very bad” sleep quality in the preceding month. The average sleep duration was 6.64 hours (SD±2.1) and average sleep efficiency was 86.6% (SD±31.7%). The majority (58.8%) of respondents reported at least mild EDS, with 15.0% reporting moderate or severe EDS (Table 2).

**Table 2:** Prevalence of sleep-related parameters in the survey respondents (total n=10,106).

Variable		Number	Mean (SD)	Proportion, %
Subjective sleep quality^1^	Very good	1,592		15.8
	Fairly good	4,674		46.2
	Fairly bad	2,861		28.3
	Very bad	979		9.7
Sleep latency^1^	0-5 minutes	716		7.1
	5-15 minutes	2,751		27.2
	15-30 minutes	2,979		29.5
	>30 minutes	3,660		36.2
Sleep duration (h)		10,106	6.64 (2.1)	
Sleep efficiency (%)		9,9612	86.6 (31.7)	
Epworth sleepiness scale^3^	Normal	3,724		36.8
	Mild	4,871		48.2
	Moderate	959		9.5
	Severe	552		5.5

**Notes:**^1^Reported by respondents;^2^n=9,961 could be calculated;^3^ 0-10 normal, 11-12 mild, 13-15 moderate, 16-24 severe excessive daytime sleepiness.

### Prevalence of bedtime technology use

Only 416 (4.1%) of respondents did not have any form of technology in their bedroom (Table 3). 95.1% of respondents had a smartphone in their bedroom, while 21.4%, 32.9%, 17.9%, 2.6%, and 7.5% had a tablet, computer, television, radio, or music player in their bedrooms, respectively. The proportion of individuals with ≤1 or ≥2 devices in their bedrooms was similar at ~50%. The vast majority of respondents reported regularly using their smartphones when they should have been sleeping (80.7%), while fewer respondents used tablet computers (10.3%) or other devices (all <10%).

**Table 3:** Bedtime technology use by the survey respondents (total n=10,106). Frequency of electronic device use was binarized into “infrequent” (never, rarely, or a few nights a month) and “frequent” (a few nights a week, every or almost every night).

Variable		Number	Percentage
**Technology in bedroom (n=10,106)**	Smartphone	9,606	95.1
	Tablet	2,162	21.4
	Computer	3,329	32.9
	TV	1,811	17.9
	Radio	258	2.6
	Music player	758	7.5
**Number of devices (n=10,106)**	≤1	4,994	49.4
	≥2	5,112	50.6
**Frequency of smartphone use (n=9890)**	Infrequent	1,905	19.3
	Frequent	7,985	80.7
**Frequency of tablet use (n=9795)**	Infrequent	8,789	89.7
	Frequent	1,006	10.3
**Frequency of computer use (n=9816)**	Infrequent	8,899	90.7
	Frequent	917	9.3
**Frequency of TV use (n=9805)**	Infrequent	9,025	92.0
	Frequent	780	8.0
**Frequency of radio use (n=9790)**	Infrequent	9,655	98.6
	Frequent	135	1.4
**Frequency of music player use (n=9789)**	Infrequent	9,288	94.9
	Frequent	501	5.1

### Associations between demographic and sleep parameters and bedtime technology use

Multivariable logistic regression models were constructed to examine associations between the number of electronic devices in the bedroom and the frequency of their use and sleep-related parameters. Associations between the number of electronic devices in the bedroom and the frequency of their use and demographic parameters are shown in Supplementary Table 1. Of interest, regular device users (those using devices a few nights a week, every or almost every night) or users with multiple devices in the bedroom were, in general, more likely to be male and divorced or single rather than female and married.

**Figure 1:**
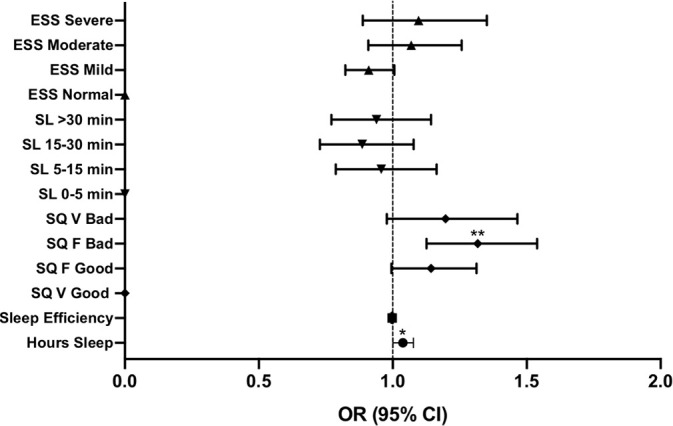
Binary logistic regression examining the association between the number of devices in the bedroom (≤1 or ≥2) and sleep parameters: number of hours slept, sleep efficiency, sleep quality (SQ; V=very, F=fairly), sleep latency (SL), and excessive daytime sleepiness (ESS). Points represent odds ratios (OR), error bars represent 95% confidence intervals (CI).

When controlling for these demographic variables (age, gender, BMI, marital status, and sleeping medication use), the number of devices in the bedroom had little effect on sleep quality parameters and EDS, with a small but significant effect on the number of hours slept (OR 1.04, 95% CI: 1.00-1.08; *p*=0.045; Figure 1) and perceived sleep quality (OR 1.32, 95% CI: 1.13-1.54; *p*=0.001 for “fairly bad” sleep quality; Figure 1).

**Figure 2:**
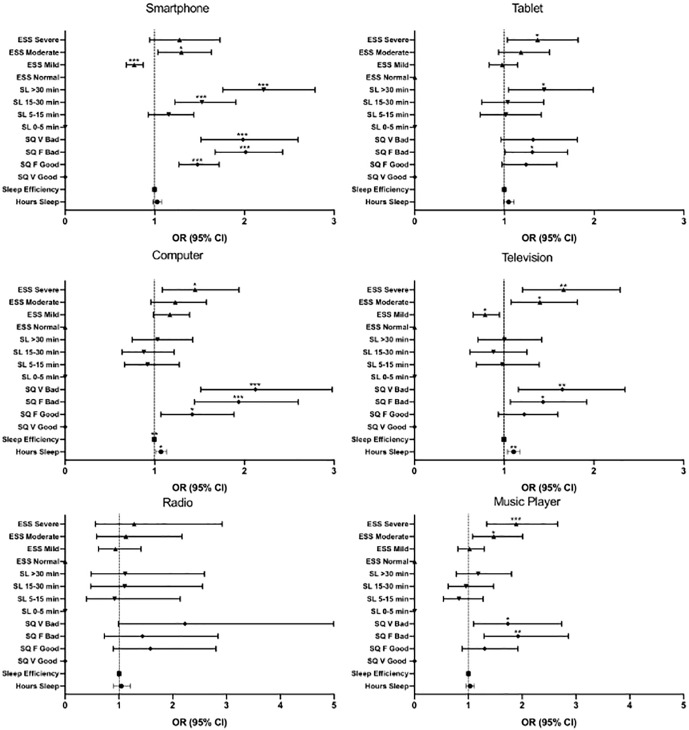
Binary logistic regression examining the association between frequent and infrequent device use in the bedroom and sleep parameters: number of hours slept, sleep efficiency, sleep quality (SQ; V=very, F=fairly), sleep latency (SL), and excessive daytime sleepiness (ESS). Points represent odds ratios (OR), error bars represent 95% confidence intervals (CI).

However, regular bedtime use (a few nights a week, every or almost every night) of individual electronic devices had greater effects on sleep-related parameters (Figure 2). Regular use of almost all devices was associated with reduced, and generally “bad” or “very bad,” sleep quality (ORs 1.32-2.12); the risk of very bad sleep quality was particularly pronounced with regular smartphone (OR 1.98, 95% CI: 1.52-2.60; *p*<0.0001) or computer use (OR 2.12, 95% CI: 1.51-2.99; *p*<0.0001) in the bedroom when the respondent would normally have been sleeping. Only smartphone or tablet use were associated with increased sleep latency, with regular smartphone use conferring a two-fold risk of taking >30 minutes to fall asleep (OR 1.98, 95% CI: 1.51-2.60) and regular tablet use conferring an ~1.5-fold risk of taking >30 minutes to fall asleep (OR 1.44, 95% CI: 1.05-1.99). The effects of regular electronic device use on EDS were modest, with smartphone, tablet, computer, and television use all associated with a 1.3-1.7-fold risk of moderate to severe EDS and regular use of a music player conferring a slightly higher risk (OR 1.89, 95% CI: 1.34-2.66; *p*<0.0001).

## DISCUSSION

Here we present new data on the prevalence of electronic device use at bedtime, sleep quality, and EDS in a large representative sample of the adult Saudi Arabian population and their inter-relationships. Similar to other populations and ethnicities, the results indicate a high burden of sleep dysfunction in Saudi Arabia: 38.0% of respondents self-reported fairly or very bad sleep quality and 15.0% moderate or severe EDS, which has previously been reported to affect between 3 and 38% of the population depending on the definition of EDS and the methodology used^23^. The average sleep duration of the sample was 6.6 hours, less than reported in the Australian 2016 Sleep Health Foundation National Survey (seven hours^24^) and under the 7-9 hours recommended for adults by the National Sleep Foundation^25^.

The landscape of technology use has evolved rapidly over the last few years, and contemporary data on the prevalence of technology in the adult bedroom are scare. This study found that electronic devices in the bedroom were almost ubiquitous, with ~95% of the sample reporting at least one electronic device in their bedroom, usually a smartphone, which was used regularly by four fifths of respondents during the time that they should have been sleeping. Of course, smartphone ownership is now extremely common; in advanced economies such as Saudi Arabia, >90% of people own smartphones^26^, and in a 2016 study of 844 Flemish adults, half of respondents owned a smartphone^9^. Specifically with respect to smartphone use in the bedroom, even in 2011, the National Sleep Foundation’s 2011 Sleep in America poll reported that 95% of respondents used electronic devices at least a few nights a week within the hour before bed, although televisions were the most popular device at that time^10^. Bhat et al. (2018)^7^ reported that 70% of a sample of 855 hospital employees used social media while in bed, while very recently Lastella et al. (2020)^11^ conducted telephone interviews in 1,225 adults and established that 42% reported using electronic devices in bed after lights out. In a recent study, 90% of highly selected adults working in a healthcare institution in Saudi Arabia reported using their smartphones at bedtime^6^, consistent with the current results. These data provide new insights into the very high prevalence of electronic device use in the bedroom in adults in a developed country, a result likely to be mirrored in similarly developed countries where nearly the entire adult population owns a smartphone.

There have only been a few studies of bedtime technology use and sleep quality in adult populations^6,7,9,11,12^, with the largest study representing 1,225 participants^11^; the current study is therefore the largest to examine this topic. The current data showed that the number of devices in the bedroom had little association with sleep quality parameters. However, regular use of almost all devices was associated with reduced subjective sleep quality. The data showing that there was a particularly pronounced risk of very bad sleep quality with regular smartphone or computer use are consistent with a very recent study, showing that duration of electronic device used was associated with poorer sleep quality in a general population of adults as determined using the same scale (very good, fairly good, fairly bad, or very bad)^11^. Furthermore, in their study of adult healthcare workers in Saudi Arabia, Alshobaili et al. (2019)^6^ established a dose-dependent relationship between the time spent using a smartphone at bedtime and risk of poor sleep quality as measured using the Pittsburgh sleep quality index (PSQI), with odds ratios ranging from 2.2 for 15-30 minutes of use to 7.5 for >60 minutes of use.

In the current study, smartphone and tablet use but no other device use conferred a 1-5-2-fold risk of longer sleep latency (>30 minutes), an association not detected in other recent studies of electronic device use and sleep^6,7,9,11,12^. However, the National Sleep Foundation’s 2011 Sleep in America poll similarly found that use of “active” electronic devices such as computers and mobile phones in the hour before bed impacted sleep latency, while “passive” devices such as televisions and music players did not^10^. Smartphones and tablets are important sources of short wavelength blue light that has been shown to suppress melatonin after only two hours of exposure and lead to sleep dysfunction, and recognizing this many have recently been equipped with “nighttime modes” to reduce blue light emission^13,27^. Indeed, in an interventional study, wearers of a blue light shield worn two hours before sleep had significantly reduced sleep latency compared to controls^28^. Smartphone and tablet users, who are likely to hold these portable devices close to their eyes and receive high levels of blue light, may be particularly adversely affected by this phenomenon. Furthermore, the content viewed on smartphones and tablets is likely to be more stimulating than that received aurally (such as music and radio). Overall, these types of active device are likely to expose individuals to all three modes of sleep disruption, namely light exposure, sleep displacement, and increased arousal. Rather than solely using nighttime modes to reduce the chances of sleep disruption, optimal sleep hygiene might be to not to use these devices at all before sleeping.

This study detected only modest effects of bedtime technology use on EDS. Bhat et al. (2018)^7^ similarly used the Epworth sleepiness scale to assess EDS but found no association between high electronic social media use after lights out and daytime sleepiness^7^, although this might be explained by the study specifically examining social media use rather than electronic device use in general. Although they used a different instrument to assess daytime sleepiness, Saling et al. (2016)^12^ similarly found that using a phone after lights out made a small but significant contribution to daytime sleepiness. Taken together with the current results, bedtime electronic device use may well contribute to daytime sleepiness, but whether this is a result of sleep displacement rather than the effects of blue light exposure or stimulating content still requires further clarification.

Given that the data on nighttime technology use and sleep in adults are relatively scarce, it is worth examining the current data with more extensive literature in children and adolescents. Our data are consistent with studies examining screen-based electronic device use in children and adolescents, with a meta-analysis of the literature (n=20 studies) showing that bedtime media device use is associated with inadequate sleep quantity (OR 2.17, 95% CI: 1.42-3.32; *p*<0.001), poor sleep quality (OR 1.46, 95% CI: 1.14-1.88; *p*=0.003), and excessive daytime sleepiness (OR, 2.72, 95% CI: 1.32-5.61; *p*=0.007)^8^. Although it has been suggested that the relationships between technology and sleep disturbances in adults and children differ^9^, our data suggest that bedtime electronic device use probably similarly impacts adults, at least in terms of sleep quality and EDS.

This study has a number of limitations. This was a self-reporting survey, with its inherent limitation of recall bias, and many people tend to overestimate sleep latency and underestimate total time asleep^29^. Like all cross-sectional surveys, no conclusions about causality can be drawn. The available survey sample was from those enrolled in the STC database, and this may have introduced selection bias, particularly since these individuals already use communication technologies. There may also have been responder bias, since prospective participants were told about the purpose of the research. Nevertheless, the young median age of the sample (28 years vs. 29.9 years) and high obesity prevalence (31.2% vs. 35.4%) are highly consistent with key sociodemographic statistics for Saudi Arabia^30^, increasing confidence that the survey is representative of the wider population. Other confounders that might affect sleepiness such as caffeine intake, shift-work, and comorbidities were not included in the analysis and might reduce or abrogate these effects in progressively adjusted outcome models^31,32^. Approximately 10% of the demographic data were missing or incomplete; however, there was very little missing sleep parameter or technology use data, providing confidence in the results. Although the study is strengthened by having looked at individual device usage, we did not establish the temporal relationship between device use and bedtime routines or the duration of their use, which would have been useful for examining dose effects. Although the study was conducted in a single country and therefore the results will be subject to cultural and geographical biases, Saudi Arabia is nevertheless an economically developed country with similar adoption of technology to other high-income countries, so the results are likely to be generalizable, at least in terms of the broad conclusion that technology use at bedtime impacts sleep quality and sleepiness.

This is the largest study to examine the relationships between bedtime technology use, EDS, and sleep quality in adults at the population-wide scale. Despite the limitations of self-reporting surveys and the potential for selection bias, this study provides insights into not only the prevalence of sleep problems but also bedtime technology use and their relationships. Like in other countries, there is a high burden of sleep-related dysfunction in Saudi Arabia, which given its impact on health, waking function, and short- and long-term wellbeing, constitutes a public health priority. Our data strengthen the currently limited evidence that electronic device use impacts sleep quality. Given that sleep hygiene advice issued by healthcare providers does not consistently include information on electronic device use, there is a need to update all sleep hygiene advice to include limiting electronic device use in the bedroom and, if their use is absolutely necessary, to apply nighttime modes to reduce blue light emission.

## Supplementary Information The questionnaire used in this study.

**Table:**

**SECTION 1: Thinking about your sleep over the last month…**
1. How long does it take you to fall asleep?
0-5min, 5-15min, 15-30min, or more than 30min.
2. How many hours of sleep do you normally get (excluding naps)?
3. On a typical ‘working’ day, what time would do you go to bed?
4. On a typical ‘working’ day, what time would do you go to sleep?
5. During the past month, how would you rate your sleep quality overall?
Very good, fairly good, fairly bad, or very bad.
**SECTION 2: About your sleepiness during the day…**
6. How likely are you to doze off or fall asleep sitting and reading?
Would never doze/slight chance of dozing/moderate chance of dozing/high chance of dozing.
7. How likely are you to doze off or fall asleep watching TV?
Would never doze/slight chance of dozing/moderate chance of dozing/high chance of dozing.
8. How likely are you to doze off or fall asleep sitting, inactive in a public place (e.g., a theatre or a meeting)?
Would never doze/slight chance of dozing/moderate chance of dozing/high chance of dozing.
9. How likely are you to doze off or fall asleep as a passenger in a car for an hour without a break?
Would never doze/slight chance of dozing/moderate chance of dozing/high chance of dozing.
10. How likely are you to doze off or fall asleep lying down to rest in the afternoon when circumstances permit?
Would never doze/slight chance of dozing/moderate chance of dozing/high chance of dozing.
11. How likely are you to doze off or fall asleep sitting and talking to someone?
Would never doze/slight chance of dozing/moderate chance of dozing/high chance of dozing.
12. How likely are you to doze off or fall asleep sitting quietly after a lunch without alcohol?
Would never doze/slight chance of dozing/moderate chance of dozing/high chance of dozing.
13. How likely are you to doze off or fall asleep in a car, while stopped for a few minutes in the traffic?
Would never doze/slight chance of dozing/moderate chance of dozing/high chance of dozing.
**SECTION 3: About your electronic device use at bedtime…**
14. Do you have a smartphone in your bedroom?
Yes/No (skip next question if no).
15. How often do you use the smartphone in bed when you would normally be sleeping?
Never/rarely/a few nights a month/a few nights a week/every or almost every night.
16. Do you have a tablet in your bedroom?
Yes/No (skip next question if no).
17. How often do you use the tablet in bed when you would normally be sleeping?
Never/rarely/a few nights a month/a few nights a week/every or almost every night.
18. Do you have a music player in your bedroom?
Yes/No (skip next question if no).
19. How often do you use the music player in bed when you would normally be sleeping?
Never/rarely/a few nights a month/a few nights a week/every or almost every night.
20. Do you have a computer/laptop in your bedroom?
Yes/No (skip next question if no).
21. How often do you use the computer/laptop in bed when you would normally be sleeping?
Never/rarely/a few nights a month/a few nights a week/every or almost every night.
22. Do you have a TV in your bedroom?
Yes/No (skip next question if no).
23. How often do you use the TV in bed when you would normally be sleeping?
Never/rarely/a few nights a month/a few nights a week/every or almost every night.
24. Do you have a radio in your bedroom?
Yes/No (skip next question if no).
25. How often do you use the radio in bed when you would normally be sleeping?
Never/rarely/a few nights a month/a few nights a week/every or almost every night.
**SECTION 4: About you…**
17. What is your gender?
Male/Female.
18. How old are you?
19. How tall are you (in cm)
20. How much do you weigh (in kg)?
21. What is your marital status?
Married/common law, single, separated, divorced, or widowed.
22. Over the past one month, how many times did you take medication (with or without a prescription) to help you sleep?

**Supplementary Table 1:** Binary logistic regression examining the association between number of devices or frequent and infrequent device use in the bedroom and demographic parameters expressed OR (95% CI; p=value). Dash (-) denotes reference group.

Parameter		Number of devices (≤1 or ≥2	Smartphone	Tablet	Computer	Television	Radio	Music player
**Age**		**1.01 (1.00-1.01; 0.04)**	**0.95 (0.95-0.96; p<0.0001)**	0.99 (0.98-1.00; 0.20)	**0.97 (0.96-0.99; p<0.0001)**	**1.03 (1.02-1.04; p<0.0001)**	**1.07 (1.06-1.09; p<0.0001)**	**0.95 (0.93-0.97; p<0.0001)**
**Gender**	**Female**	-	-	-	-	-	-	-
	**Male**	**1.29 (1.34-1.65; p<0.0001)**	**1.31 (1.15-1.50; p<0.0001)**	**1.44 (1.21-1.71; p<0.0001)**	**1.48 (1.23-1.78; p<0.0001)**	**0.52 (0.43-0.63; p<0.0001)**	0.84 (0.55-1.30; 0.44)	**1.46 (1.13-1.88; 0.004)**
**Marital status**	**Married**	-	-	-	-	-	-	-
	**Divorced**	**3.04 (2.32-3.97; p<0.0001)**	**1.54 (1.08-2.21; 0.02)**	**2.71 (1.89-3.89; p<0.0001)**	**3.50 (2.27-5.39; p<0.0001)**	**1.99 (1.26-3.12; 0.003)**	1.07 (0.38-3.02; 0.90)	**2.08 (1.03-4.18; 0.04)**
	**Single**	**4.87 (4.26-5.56; p<0.0001)**	1.16 (0.99-1.37; 0.07)	**1.81 (1.45-2.26; p<0.0001)**	**3.16 (2.42-4.12; p<0.0001)**	**2.43 (1.90-3.11; p<0.0001)**	1.39 (0.80-2.40; 0.24)	**2.28 (1.56-3.32; p<0.0001)**
	**Widowed**	1.61 (0.90-2.88; p=0.11)	0.96 (0.51-1.82; 0.90)	2.03 (0.83-4.94; 0.12)	1.70 (0.40-7.25; 0.47)	1.31 (0.45-3.79; 0.62)	2.32 (0.80-6.69; 0.12)	Sample too small
**Sleeping medication use**	**Never**	-	-	-	-	-	-	-
	**Several days**	1.1 (0.95-1.29; 0.21)	0.93 (0.75-1.13; 0.46)	**1.32 (1.07-1.65; 0.009)**	0.89 (0.69-1.14; 0.35)	**1.27 (0.99-1.63; 0.06)**	0.85 (0.43-1.67; 0.64)	1.27 (0.94-1.71; 0.13)
	**Over half of days**	1.34 (0.98-1.84; 0.07)	0.93 (0.60-1.43; 0.73)	0.94 (0.60-1.50; 0.81)	1.13 (0.73-1.73; 0.59)	**1.71 (1.11-2.65; 0.02)**	1.32 (0.40-4.31; 0.65)	**1.69 (1.02-2.78; 0.04)**
	**Nearly every day**	1.06 (0.75-1.50; 0.75)	1.06 (0.67-1.68; 0.81)	**1.68 (1.07-2.63; 0.02)**	1.53 (0.94-2.51; 0.09)	**1.79 (1.10-2.92; 0.02)**	1.81 (0.70-4.68; 0.22)	1.35 (0.68-2.66; 0.39)
